# Expression, purification, and contaminant detection for structural studies of *Ralstonia metallidurance* ClC protein rm1

**DOI:** 10.1371/journal.pone.0180163

**Published:** 2017-07-10

**Authors:** Priyanka D. Abeyrathne, Nikolaus Grigorieff

**Affiliations:** Howard Hughes Medical Institute, Janelia Research Campus, Helix Drive, Ashburn, VA, United States of America; University of Bern, SWITZERLAND

## Abstract

Single-particle electron cryo-microscopy (cryo-EM) has become a popular method for high-resolution study of the structural and functional properties of proteins. However, sufficient expression and purification of membrane proteins holds many challenges. We describe methods to overcome these obstacles using ClC-rm1, a prokaryotic chloride channel (ClC) family protein from *Ralstonia metallidurans*, overexpressed in *Escherichia coli* (*E*. *coli*) BL21(DE3) strain. Mass spectrometry and electron microscopy analyses of purified samples revealed multiple contaminants that can obfuscate results of subsequent high-resolution structural analysis. Here we describe the systematic optimization of sample preparation procedures, including expression systems, solubilization techniques, purification protocols, and contamination detection. We found that expressing ClC-rm1 in *E*. *coli* BL21(DE3) and using n-dodecyl-β-D-maltopyranoside as a detergent for solubilization and purification steps resulted in the highest quality samples of those we tested. However, although protein yield, sample stability, and the resolution of structural detail were improved following these changes, we still detected contaminants including Acriflavine resistant protein AcrB. AcrB was particularly difficult to remove as it co-purified with ClC-rm1 due to four intrinsic histidine residues at its C-terminus that bind to affinity resins. We were able to obtain properly folded pure ClC-rm1 by adding eGFP to the C-terminus and overexpressing the protein in the Δ*acrB* variant of the JW0451-2 *E*. *coli* strain.

## Introduction

X-ray crystallography, nuclear magnetic resonance (NMR), electron crystallography, and single-particle electron cryo-microscopy (cryo-EM) have all been used to obtain high-resolution structural information from both soluble and membrane proteins. However, using these techniques to resolve the structures of membrane proteins is especially challenging. The purification process, necessary to prepare suitable samples for high-resolution structural imaging, is particularly labor-intensive for membrane proteins, due to their hydrophobicity and reduced stability in solution. In response to these challenges, work in the field has moved towards leveraging fluorescence size-exclusion chromatography (FSEC) to screen detergents and buffer conditions in a high-throughput manner [1–3]. However, producing sufficient quantities of stable protein with minimal contaminants continues to be a major bottleneck [4].

Single-particle cryo-EM has the distinct advantage that it does not require crystals to obtain 3D structural information. It can be used to solve the structure of macromolecules in more native conformations, as rapid freezing makes it possible to capture specific functional states. These advantages, combined with recent advances in detector technology and data processing algorithms, have led to a wide application of single-particle cryo-EM. Here, we describe our systematic approach to determining the optimal preparation procedure for structural analyses, cryo-EM in particular, of the prokaryotic chloride channel (ClC) protein ClC-rm1 of *Ralstonia metallidurans* (*R*. *metallidurans*). This protein is challenging for structural studies, particularly single-particle cryo-EM, because of its relatively small size (the dimer’s molecular mass is 115 kDa), and the fact that 85% of the protein is embedded in the membrane. Mass spectrometry analysis and negative stain EM revealed that samples of ClC-rm1 prepared following standard procedures included several contaminants. Therefore, we set out to optimize the expression system, solubilization and purification protocols, and contaminant detection techniques. We were able to produce high-quality, stable sample for cryo-EM while eliminating nearly all contaminants.

## Materials and methods

The general approach to preparing membrane protein for high-resolution structural studies involves: (1) cloning and expression; (2) solubilization; (3) purification; (4) detergent exchange; and (5) assessment of stability, quality, and purity. We systematically optimized each of these preparation steps.

### 1. Cloning and expression

The protein expression procedure, including plasmid, expression vectors, growth medium, expression time and temperature, was varied systematically to determine optimal conditions. We tested different vectors to express the *R*. *metallidurans* ClC gene RMET_RS02305: (1) pET30a (Invitrogen) and (2) pBAD18 (Yale University). For expression in the pET30a vector we added a C-terminal 6x(His) tag. For expression in the pBAD18 vector, we added enhanced green fluorescence protein (eGFP) and a 10x(His) tag at the C-terminus. The plasmid was then expressed in one of two different *Escherichia coli* (*E*. *coli*) strains, BL21(DE3) or JW0451-2, and grown in either Luria-Bertani (LB) or terrific broth (TB). We also tested two different expression protocols that varied in timing and temperature. In the first method, expressed protein in BL21(DE3) *E*. *coli* cells was harvested 2 h after induction with 1 mM of isopropyl-thio-β-D-galactopyranoside (IPTG) at 37°C. In the second method, protein was expressed in *E*. *coli* JW0451-2 cells, which were grown at 37°C under vigorous shaking to an OD_600_ = 0.5, cooled to 25°C, and then induced with 1% (w/v) arabinose for 12 h. In both cases, membranes were prepared as described in [5, 6].

### 2. Solubilization

Solubilization screens were conducted in 5 ml total volume mixtures with membranes diluted to 1 mg/ml and solubilized in buffer containing 300 mM NaCl, 1% (w/v) glycerol, and 20 mM Tris-HCl pH 8.0. The concentration of detergent added varied depending on type: 1.5% (w/v) DDM, 1.4% (w/v) DM, and 5% (w/v) OG. After incubation for 1 h at 4°C, soluble and insoluble fractions were separated by centrifugation at 35,000 rpm for 30 min.

Conventional protein detection methods, such as SDS-PAGE gel electrophoresis and Western blot analysis, were used to analyze the soluble fractions for protein yield and efficiency of extraction. Solubilized ClC-rm1 fractions in detergent were analyzed by NUPAGE 4–12% Bis-Tris gel electrophoresis (Invitrogen) and 1% (v/v) MES as running buffer (Invitrogen) and visualized by SimplyBlue SafeStain (Invitrogen) or Silver stain. Western blot was carried out according to the manufacturer’s instructions using the WesternBreeze Chromogenic Western Blot Immunodetection Kit (Invitrogen) and His tag antibody as primary antibody (ABGENT). The secondary antibody was an alkaline phosphate conjugated, affinity purified, anti-specific IgG (Invitrogen).

### 3. Purification

We purified solubilized fractions of ClC-rm1 that had been extracted using DDM, DM, or OG. Since ClC-rm1 carries a C-terminal histidine tag, nickel (Ni^2+^) and cobalt (Co^2+^) resins were utilized for affinity purification. Soluble fractions were separately adsorbed to Ni^2+^ and Co^2+^ resins for 1 h at 4°C, then washed twice: first in 0.1% (w/v) DDM in 295 mM NaCl, 5 mM KCl, 30 mM imidazole and 20 mM Tris-HCl pH 8.0 and subsequently in 0.1% (w/v) DDM in 295 mM NaCl, 5 mM KCl, 50 mM imidazole and 20 mM Tris-HCl pH 8.0. The protein was eluted in 0.1% (w/v) DDM in 295 mM NaCl, 5 mM KCl, 400 mM imidazole and 20 mM Tris-HCl, pH 8.0 at 4°C. The same procedure was applied to solubilized fractions extracted using DM or OG.

Eluted ClC-rm1 was subjected to pre-equilibrated 0.03% (w/v) DDM in 150 mM NaCl, 5 mM KCl, and 20 mM Tris-HCl pH 8.0 with superose 6 (initial experiments) and superdex 200 (for later experiments) size exclusion chromatography (SEC) (GE Healthcare) to remove imidazole and protein aggregates, and to minimize the detergent concentration to avoid surpassing the critical micelle concentration (CMC) of each detergent. The peak corresponding to dimeric ClC-rm1 was collected and analyzed by SDS-PAGE gel electrophoresis (to determine purity), negative stain EM and single-particle image processing (to determine purity and homogeneity), and mass spectrometry (to detect contaminants).

### 4. Detergent exchange

DDM used in sample preparation was exchanged with either DM, OG, or the amphipathic polymer, amphipol A8-35. When exchanging DDM for DM, purified ClC-rm1 in DDM was run through the Superdex 200 SEC column in buffer composed of 150 mM NaCl, 0.20% DM and 20 mM Tris-HCl pH 8.0. When exchanging DDM for OG, purified ClC-rm1 in DDM was run through the Superdex 200 SEC column in buffer composed of 150 mM NaCl, 20 mM, 1% (w/v) OG and Tris-HCl pH 8.0. When exchanging DDM for A8-35, purified ClC-rm1 in DDM was mixed with amphipol A8-35 at 1:3 (w/w) with gentle agitation for 2 h at 4°C. Detergent was removed with Bio-Beads SM-2 for 8 h at 4°C. Protein was then run through the Superdex 200 SEC column in buffer composed of 150 mM NaCl and 20 mM Tris-HCl pH 8.0. In all cases, the peak corresponding to dimeric ClC-rm1 was collected and analyzed using SDS-PAGE gel electrophoresis followed by negative stain EM and single-particle image processing to assess the purity and homogeneity of samples.

### 5. Stability, quality, and impurity assessment

We used SEC to assess the stability and quality of purified ClC-rm1 in detergent. SEC fractions of dimeric ClC-rm1 in DDM, DM, or OG were kept at 4°C for three days and then run again on a SEC column to determine long-term stability.

Electron microscope micrographs of negatively-stained ClC-rm1 were used to assess the quality and purity of the sample. Grids were prepared by applying 3 μl of ClC-rm1 (~ 40 ng/μl) onto a carbon-coated copper grid. To avoid interference with the staining of the protein, the detergent-containing buffer was removed, after ClC-rm1 was adsorbed to the carbon film, by washing the grid with eight drops of deionized water prior to staining with 1.5% (w/v) uranyl formate. The grids were then screened under low-dose conditions using a Technai T12 microscope (FEI) operating at 120 kV, and a Technai F20 microscope (FEI) operating at 200 kV. Micrographs taken on the T12 were recorded at nominal magnifications of 49,000x and 68,000x on a 4k x 4k TVIPS CMOS camera, resulting in 2.1 and 1.5 Å/pixel, respectively. Micrographs taken on the F20 were recorded at nominal magnifications of 62,000x on a 4k x 4k TVIPS CMOS camera, resulting in 1.33 Å/pixel. Single-particle image processing was carried out using IMAGIC software [7]. Micrographs with excessive drift, low contrast, and poor power spectra were removed based on visual inspection with TIGRIS (http://tigris.sourceforge.net/). 50 particles were picked manually using TIGRIS, summed and rotationally averaged to serve as a reference for correlation-based particle picking in IMAGIC. These particle images were band-pass filtered to suppress very low and high spatial frequencies, and a circular mask was applied to remove unwanted background. The particle images were then normalized to zero average density and variance of 1. Those pre-processed images underwent cycles of classification and reference-free alignment, as implemented in IMAGIC, without imposing any symmetry.

We then tested whether the purified ClC-rm1_eGFP sample was suitable for high-resolution data collection. The following specimen preparation procedure was used to make cryo-grids of ClC-rm1_eGFP. C-flat Cu grids (Protochips, Protochips, Inc) were glow discharged for 60 s at 25 mA. 3 μl of sample with a concentration of ~2 mg/ml was applied to the grid and plunged into liquid ethane using FEI Vitrobot Mark 2 (FEI Company, Hillsboro, OR) after blotting for 3 s at 4°C and ~85% relative humidity. Cryo-EM data were collected in movie mode on an FEI Krios microscope (FEI Company, Hillsboro, OR) operating in super-resolution mode with pixel size of 0.82 Å per super-resolution pixel. Each movie consisted of 75 frames collected over 23 s with an exposure per frame of 1.4 e-/Å2 as shown by Digital Micrograph (Gatan Inc., Pleasanton, CA), giving a total exposure of 100 e-/Å2. The image defocus range between ~0.8 to ~2.5 μm underfocus. The gain-corrected super-resolution movie frames were downsampled by Fourier cropping to a pixel size of 1.64 Å. The down-sampled frames were then motion-corrected and exposure filtered using Unblur [8]. The micrograph defocus was determined using CTFFIND4 [9] on non-exposure-filtered micrographs and micrographs with excessive motion, low contrast, ice contamination or poor power spectra were removed based on visual inspection using TIGRIS. 50 particles were picked manually using TIGRIS, summed and rotationally averaged to serve as a reference for correlation-based particle picking in IMAGIC. 2D classification was performed as above using IMAGIC.

The purified ClC-rm1 samples were loaded on NUPAGE 4–12% Bis-Tris Gel (Invitrogen) and visualized by SimplyBlue SafeStain (Invitrogen) or Silver stain to detect the presence of contaminants. Visible protein bands were cut out and sent to a facility for mass spectrometry analyses to confirm identity (Taplin Biological Mass Spectrometry Facility at Harvard Medical School, Boston, MA 02115).

## Results and discussion

### Choice of model system

**Fig 1:**
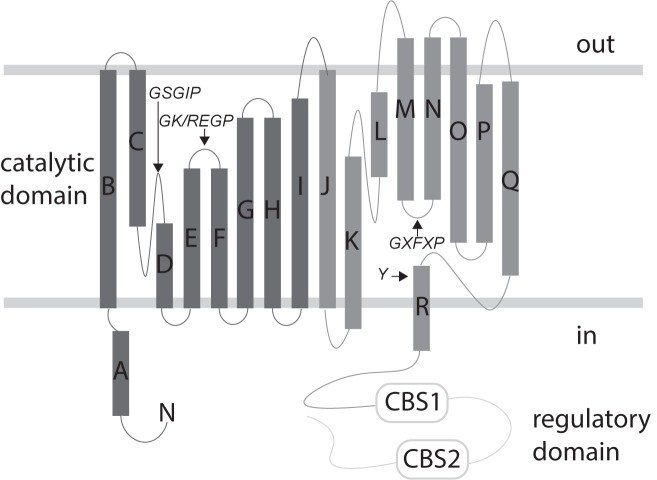
Topology of eukaryotic ClC proteins. A schematic diagram of the ClC protein containing transmembrane catalytic domain and cytoplasmic regulatory domain. The topology of the transmembrane domain is based on the ClC-ec1 protein. The 18 α helices are labeled from A-R. The two halves of the subunits are shown in two different shades of gray. The regions and sequences that contribute to the chloride selectivity filter are marked with black arrows and the respective conserved sequences are indicated (*amino acids are abbreviated with their one-letter codes*, */ (forward slash)*: *or*, *X*: *any amino acid)*. The two cytoplasmic cystathionine beta synthase (CBS) subdomains are shown in rounded rectangles (CBS1 and CBS2).

The structural organization of ClC proteins varies between prokaryotes and eukaryotes. All ClC proteins have a transmembrane catalytic domain and most (all eukaryotic types and a few prokaryotic types) have a cytoplasmic regulatory domain [10, 11]. The topology of the membrane domain of a single ClC subunit is shown in Fig 1 (based on the ClC-ec1 protein [12, 13]). In this structure, each monomer includes 18 α helices (labeled from A-R) making up two halves of a single subunit. There are two cytoplasmic CBS subdomains (CBS1 and CBS2; Fig 1, rounded rectangles). The length of the cytoplasmic domain varies among ClC proteins but in most cases appears to be involved in diverse regulatory mechanisms, including chloride transport, oligomerization, protein sorting, channel gating, and ligand binding [13–17]. However, there is a lack of more detailed information regarding the structure-function relationship of this diverse family of membrane proteins due to the challenges of preparing samples suitable for high-resolution structural imaging.

Producing high-quality protein samples in quantities suitable for imaging studies and free of contaminants is important to obtain reliable data on the structure and function of proteins. The genome sequences of many prokaryotic proteins are readily available, and they are relatively easy to express and purify compared to their eukaryotic counterparts. This makes them an ideal model system to optimize protein sample preparation techniques. The prokaryote *R*. *metallidurans* has two ClC genes RMET_RS02305 and RMET_RS02950. We chose to focus on *R*. *metallidurans* RMET_RS02305 encoding the ClC protein WP_011515329 (ClC-rm1), because this membrane protein is small (the dimer’s molecular mass is 115 kDa) and its biological function has not been characterized. ClC-rm1 has 560 amino acids and includes a transmembrane domain as well as a cytoplasmic domain.

### Optimization of expression

**Fig 2:**
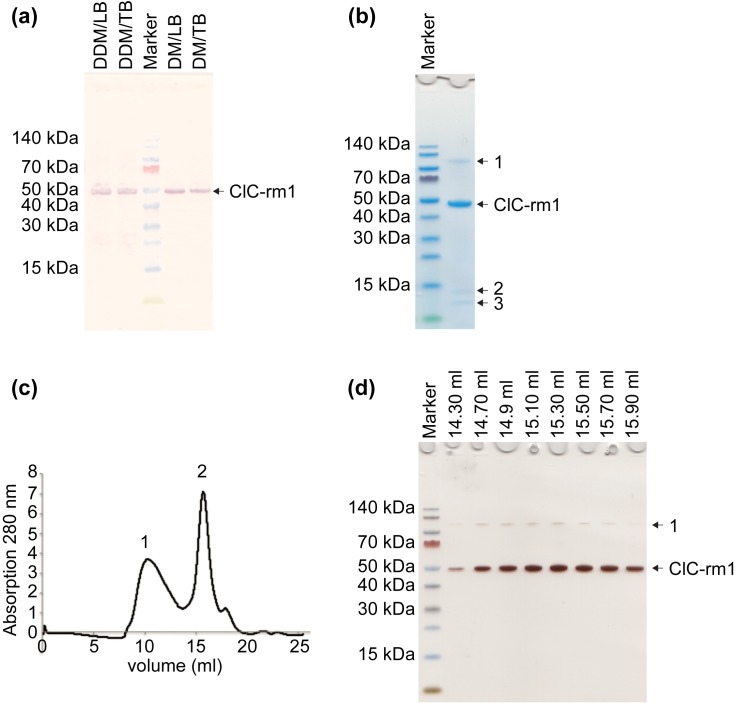
Expression and purification profiles of ClC-rm1 in pET30a. (a) ClC-rm1 was expressed using *E*. *coli* strain BL21(DE3) in either LB or TB media. Cells were harvested and the membranes isolated. Membranes were then solubilized using either DDM or DM. Western blot shows ClC-rm1 expressed in LB and TB, and solubilized membranes using DDM or DM. Lanes 1 and 2: fractions of DDM-solubilized membranes with cells grown in LB (1) and TB (2). Lane 3: molecular mass marker. Lanes 4 and 5: fractions of DM solubilized membranes with cells grown in LB (4) and TB (5). (b) ClC-rm1 was purified in DDM using Ni^2+^ resin. Lane 1: molecular mass markers. Lane 2: ClC-rm1 monomer band at ~50 kDa and three other minor bands (labeled as 1, 2 & 3). (c) Superose 6 SEC elution profile of ClC-rm1 in DDM. Peak 1 corresponds to the void volume. Peak 2 corresponds to ClC-rm1. (d) Silver stained SDS-PAGE gel of ClC-rm1 after SEC. Lane 1: molecular mass marker. The rest of the lanes correspond to the Superose 6 SEC fractions. ClC-rm1 monomer band is at ~50 kDa with only one additional band (labeled as 1).

Optimized growth medium is important for maximizing protein expression. LB broth is the most commonly used medium for protein expression on a laboratory scale as it is easy to make and readily available. Additionally, LB broth has rich nutrient contents ideally suited for growing *E*. *coli*. TB, a phosphate-buffered, rich medium, is also used extensively. TB has 0.2% (w/v) more tryptone and 5% (w/v) more yeast extract than LB, and 0.4% (v/v) glycerol as an extra carbon source. These features support fast *E*. *coli* growth and promote high cell density. In prior work [5], we found that ClC-ec1 grown in TB yielded a functionally active protein. In our optimization tests, we found that expression of ClC-rm1 in *E*. *coli* was similar in LB and TB (Fig 2A).

We next tested whether the expression system (plasmid + *E*. *coli* strain) mattered in terms of obtaining protein yield sufficient for high-resolution structural studies, including cryo-EM. We first expressed ClC-rm1 cloned into the pET30a vector using the tac promoter (induced by IPTG) in *E*. *coli* strain BL21(DE3). Under optimal expression conditions of IPTG concentration (100 mM), induction time (3 h), and temperature (37°C), we were able to obtain ~0.3 mg of purified ClC-rm1 per liter of culture (Fig 2B).

The second expression system included ClC-rm1 cloned into the pBAD18 plasmid under the P_BAD_ promoter (induced by arabinose) and grown in the *E*. *coli* strain JW0451-2. Under optimal expression conditions, i.e., 0.16% (w/v) arabinose, 18 h induction time at 25°C, we were able to obtain ~0.5 mg of purified ClC-rm1_eGFP per liter of culture.

### Selection of detergents

Membrane proteins maintain their position through hydrophobic interactions between the hydrocarbon chains of the lipids and the hydrophobic domains of the proteins. Detergents solubilize the proteins by breaking those interactions. Membrane proteins are classified as either integral or peripheral. Integral membrane protein contain one or more membrane-spanning domains, usually involving hydrophobic interactions with the lipid interior of the bilayer and probably also ionic interactions with the polar head groups of the phospholipids. In comparison, peripheral membrane proteins do not interact with the hydrophobic core of the phospholipid bilayer, but instead can bind to the membrane indirectly, via interactions with integral membrane proteins, or directly via interactions with lipid polar head groups. Therefore, individual membrane proteins have different solubilization requirements, especially regarding detergent concentration, pH, and salt concentration. Hence, it is important to test a wide range of detergents.

Charged (anionic, cationic), nonionic, and zwitterionic detergents are available to solubilize membrane proteins [18–23]. Detergents are categorized as “harsh” or “mild” according to their tendency to denature proteins [24]. In general, charged detergents are harsher than uncharged detergents, and those with larger head groups and longer hydrophobic tails are milder than those with smaller head groups and shorter tails [24]. Commonly used detergents for structural and functional studies of membrane proteins are n-dodecyl-β-D-maltopyranoside (DDM), n-octyl-β-D-glucopyranoside (OG), n-decyl-β-D-maltopyranoside (DM), n-dodecyl-N,N-dimethylamine-N-oxide (LDAO), and lauryl maltose neopentyl glycol (LMNG), CHAPS, Triton-X100 and digitonin. Of these, DDM, OG, DM, LMNG, Triton-X100 and digitonin are non-ionic detergents, and LDAO and CHAPS are zwitterionic detergents. Previous studies have shown that *E*. *coli* ClC protein ClC-ec1 can be purified in a functional state using DM [25] (S1 Fig). Here we compared results using three non-ionic detergents, DM, DDM, and OG.

We monitored solubilization efficiency using SDS-PAGE and Western blotting. We compared extraction efficiency across different detergent types and expression conditions (i.e., one of the two plasmid vectors, one of the two *E*. *coli* strains, grown in either TB or LB media; see Fig 2A for an example comparing expression levels of ClC-rm1 in the pET30a vector using the BL21(DE3) *E*. *coli* strain). Overall, Western blot analysis indicated that, OG was not as efficient as either DDM or DM.

### Optimization of purification

#### Detergent concentration optimization

Following solubilization, the protein samples were purified. Detergent type and detergent concentration must be considered in this step, as well as the specific affinity chromatography protocol. Above the CMC, detergent monomers assemble into micelles that surround the hydrophobic regions of membrane proteins, keeping them in solution. During solubilization, the detergent disintegrates the lipid-bilayer, resulting in mixed micelles of protein/detergent, protein/detergent/lipid, lipid/detergent, or micelles containing detergent alone. This is advantageous for solubilization, but the micelle around the hydrophobic surface of the membrane protein and free micelles in solution add featureful background to the images that make single-particle image processing more difficult. Thus, the ideal detergent concentration is just above the CMC, i.e., high enough to prevent protein aggregation and low enough to avoid confounds during the structural imaging process. We systematically varied the detergent concentration at or above the CMC in order to optimize this balance (please see Materials and Methods section for details) and found that the best results were obtained with 0.03%, 0.2% and 1.0% (w/v) for DDM, DM and OG.

#### Affinity chromatography

To allow for purification via affinity chromatography, affinity tags are expressed along with the protein. Histidine tags (hexa-histidine, octa-histidine, or deca-histidine) are the most widely used affinity tags, allowing for variable binding strength depending on the length of the tag used. Adding a histidine tag to the membrane protein makes it possible to rapidly purify samples using Ni^2+^ or Co^2+^ affinity chromatography.

For these studies, ClC-rm1 was expressed with a C-terminal 6x(His) tag. We compared the efficacy of Ni^2+^ versus Co^2+^ affinity chromatography in purifying ClC-rm1 solubilized with either DM or DDM detergent. DM-solubilized ClC-rm1 did not bind to either the Ni^2+^ or Co^2+^ resins. DDM-solubilized protein bound with very low affinity to Co^2+^ resin but showed strong affinity to Ni^2+^ resin. After two washing steps with increasing amounts of imidazole (to reduce the non-specific binding), ClC-rm1 was eluted from the Ni^2+^ resin with 300 mM NaCl and 400 mM imidazole. SDS-PAGE analysis of the eluted ClC-rm1 indicated one major band and three minor bands (Fig 2B). The major band observed at ~50 kDa corresponds to the ClC-rm1 monomers and the band at ~100 kDa (Fig 2B, band “1”) corresponds to ClC-rm1 dimers that are resistant to the denaturation conditions of SDS-PAGE. The two remaining bands in Fig 2B (“2” at ~ 13 kDa and “3” at ~14 kDa) reflect proteins that are non-specifically bound to the Ni^2+^ resin and are, therefore, possible contaminants.

#### Single-particle EM analysis to determine sample purity

ClC-rm1 is a small membrane protein with more than 85% of its structure embedded in the membrane. As such the detergent micelle surrounding the transmembrane domain covers most of the protein surface, making it difficult to distinguish ClC-rm1 proteins from background contaminants. Negative stain EM is commonly used to monitor homogeneity and/or heterogeneity of purified protein samples to inform the optimization of protein purification procedures. We used negative staining and single-particle image processing together with mass spectrometry to assess the quality and purity of purified ClC-rm1, with an eye towards sample optimization for cryo-EM. Working with the ClC-rm1 protein purified using the DDM/Ni^2+^ resin protocol, we used a Superose 6 column to remove imidazole and lower the DDM concentration (Fig 2C). SEC of ClC-rm1 had a large void volume peak (labeled as 1 in Fig 2C). The peak corresponding to the size of dimeric ClC-rm1 (labeled as 2 in Fig 2C) was collected and analyzed using SDS-PAGE. The resulting gel contained two bands (Fig 2D). As before, the band observed at ~50 kDa corresponds to the ClC-rm1 monomers, and the band at ~100 kDa corresponds to denaturation-resistant ClC-rm1 dimers.

**Fig 3:**
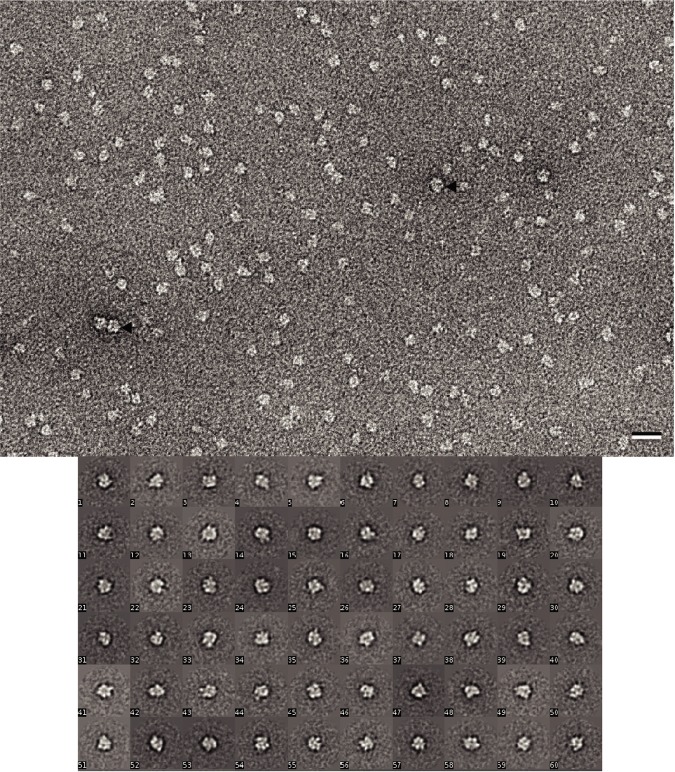
Micrographs of negatively stained ClC-rm1 in DDM. (a) DDM-solubilized ClC-rm1 particles are monodisperse. The size of some particles are different from the rest (highlighted by black arrowheads). Scale bar 20 nm. (b) Representative single-particle class averages.

We used negative stain EM to further assess sample quality. To avoid interference with the staining of protein, the detergent was removed from ClC-rm1 adsorbed to the carbon film by washing the grid with eight drops of miliQ water prior to staining with uranyl formate. Negative stain micrographs of ClC-rm1 in DDM showed monodisperse particles with a few instances of highly stained, variably sized particles that were likely contaminants (Fig 3A, black arrowheads). Representative class averages are shown in Fig 3B. These reveal a relatively round shape with a diameter of ~112 Å, consistent with the size of ClC-rm1 dimers [12]. More structural details could not be seen in the 2D classes, perhaps due to the DDM detergent belt. However, this single-particle analysis does indicate that ClC-rm1 protein samples prepared in this way are suitable for cryo-EM.

### Detergent exchange of purified ClC-rm1 in DDM

Minimizing the micelle size that surrounds the hydrophobic region of a membrane protein is important for single-particle EM, in particular when imaging small membrane proteins that are mostly embedded in the membrane. Although DDM detergent worked to provide the highest yield of ClC-rm1 during purification, it has a larger micelle size relative to DM or OG. Thus, we performed a detergent exchange experiment to test whether DDM could be replaced by a detergent that forms smaller micelles, such as DM or OG. We also tested amphipol A8-35, one of a special class of amphipathic polymer that carries hydrophobic and hydrophilic groups and is effective at keeping membrane proteins water soluble prior to imaging [26].

**Fig 4:**
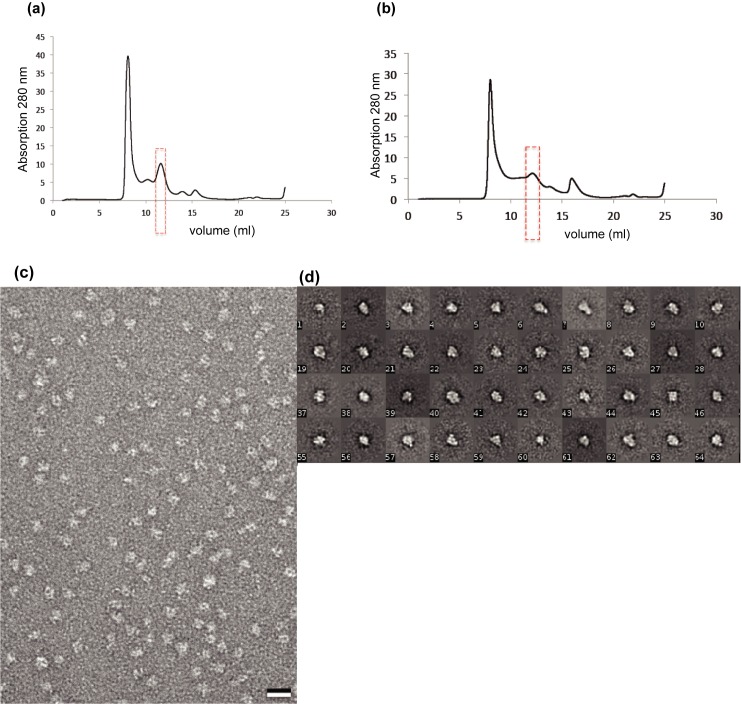
Superdex 200 SEC profile of detergent exchange. Purified ClC-rm1 in DDM was exchanged to (a) DM or (b) OG. In both cases, particles were monodisperse. (c) Micrograph of negatively stained ClC-rm1 in OG. Scale bar 20 nm. (d) Representative class averages of ClC-rm1 in OG.

For these experiments, ClC-rm1 was solubilized and purified in DDM, as described above. Then, DDM was exchanged via SEC pre-equilibriated with buffer containing either DM or OG. Since DDM has a lower CMC compared to DM and OG some DDM may have remained after the exchange. However, for single particle images, removal of the majority of DDM is sufficient. To determine whether the detergent exchange affected the protein sample quality, we compared SEC profiles before and after the exchange. SEC revealed a similar peak profile following DDM replacement with DM (Fig 4A). However, when DDM was replaced with OG, there was a dramatic drop in peak height (Fig 4B). One possible explanation for the reduced peak height is that the protein becomes less stable and begins to aggregate in the presence of OG. We also observed a shift in the retention time of the eluted protein, likely due to the change in micelle size. Negatively stained DM- and OG-exchanged fractions contained monodisperse particles, except for a few particles that varied significantly in size and the extent to which they accumulated stain. We observed similar size variations in the DDM sample. OG exchanged samples were further analyzed using single-particle image processing. 18,322 particles were selected and classified into 400 classes. Representative class averages revealed a round shape (Fig 4D).

**Fig 5:**
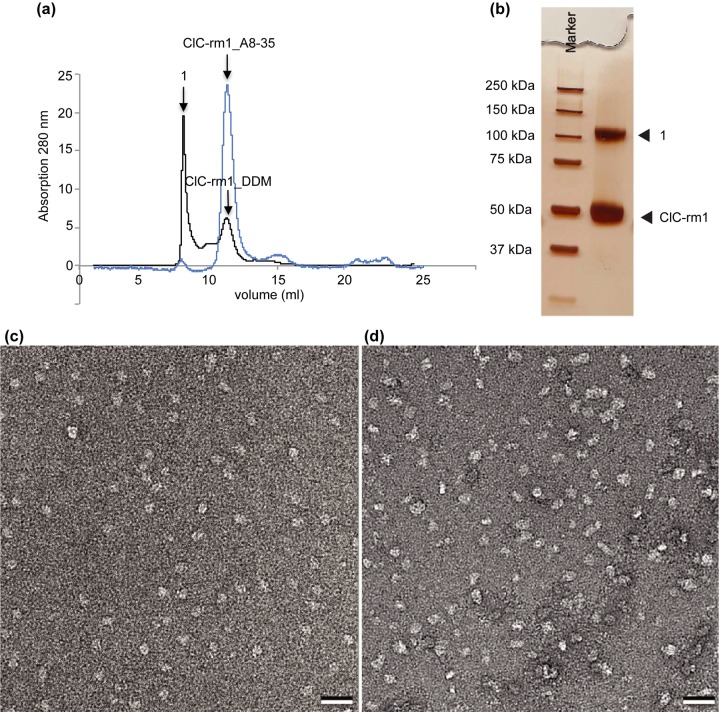
ClC-rm1 purified in DDM and exchanged with amphipol A8-35. (a) Elution profile of ClC-rm1 in DDM from Superdex 200 SEC column (black line). SEC profile showed a large void volume peak of ClC-rm1 in DDM (labeled as 1) as well as a peak corresponding to the ClC-rm1 in DDM. ClC-rm1 in A8-35 has a symmetric peak with a very small void volume peak (highlighted in blue). (b) Silver stained SDS-PAGE gel of ClC-rm1 in A8-35 after SEC. Lane 1: molecular mass marker. Lane 2: ClC-rm1 in A8-35, ClC-rm1 monomer band at ~50 kDa with only one additional band (labeled as 1). (c) Micrograph of negatively stained ClC-rm1 in DDM used as initial materal for the A8-35 exchange experiment. (d) Micrograph of negatively stained ClC-rm1 in A8-35. Scale bar 20 nm.

**Fig 6:**
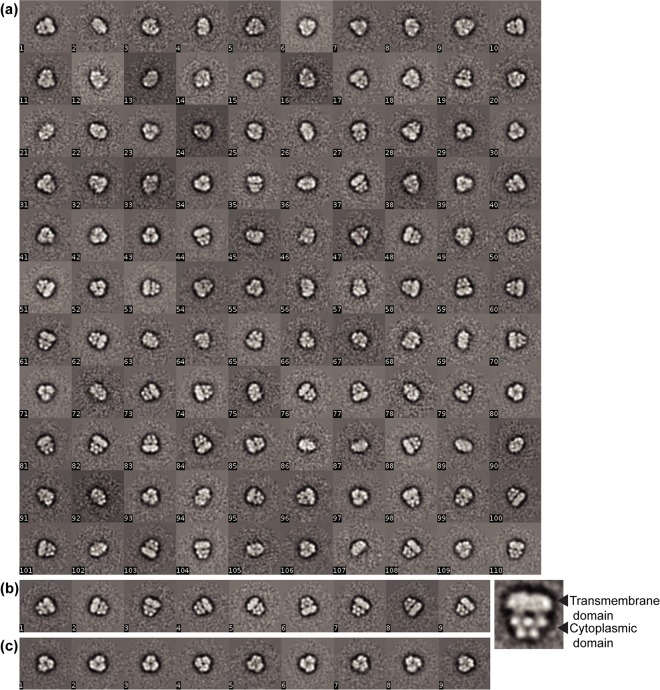
(a) Representative classes of ClC-rm1 in A8-35. (b) Classes with clear transmembrane and cytoplasmic domains were extracted from the original classes. (c) Classes with trimeric structure were extracted from the original classes.

Recently, high-resolution structures of a number of different membrane proteins were obtained using cryo-EM from samples containing amphipol A8-35 [27–30]. Amphipols self-assemble into well-defined particles comprising an average of four macromolecules,[31] e.g., for A8-35, which has a molecular mass of 9–10 kDa. They also exhibit a high affinity for hydrophobic particles. Because of these unique properties, using amphipols to surround membrane proteins results in minimal extra density around the proteins’ hydrophobic domains compared to detergents. Although ClC-rm1 in DDM showed monodisperse particles on negative-stained micrographs (Fig 3A), we decided to test whether amphipol A8-35 would reveal more detailed features within the ClC-rm1 transmembrane domain. ClC-rm1 in A8-35 was monodisperse, as evidenced by the single peak following SEC (Fig 5A). There was also no evidence of an aggregation peak (Fig 5A). Thus, exchanging DDM for A8-35 appeared to maintain sample purity and stability. Analysis of ClC-rm1 in A8-35 on SDS-PAGE revealed two bands, again similar to other detergents (Fig 5B). However, the band around 50 kDa was predominant. Indeed, negatively-stained samples showed that the sample was not as monodisperse (Fig 5D) as ClC-rm1 in DDM (Fig 5C). 6056 particles were selected for 2D classification into 300 classes. Representative classes are shown in Fig 6, revealing different sizes and shapes. They reveal features that are distinct from those seen in the ClC-rm1 classes obtained with material solubilized in DDM and OG, and the particle size appears to be larger than ClC-rm1 (150 Å height x 150 Å width; Fig 6B). Some classes showed trimeric structures (Fig 6C) and further investigation showed that this larger particle likely corresponds to AcrB [32]. These results therefore indicate that the protein band (Figs 1B, 1D and 5B, labeled as “1”) on SDS-PAGE is consistent with the molecular mass of dimeric ClC-rm1, it likely contains contaminant proteins co-migrating with dimeric ClC-rm1. It is also worth noting that AcrB and the trimeric protein structure shown in Fig 6C are likely present in the same SEC elution fractions as ClC-rm1. The significant contamination of ClC-rm1 with AcrB when exchanging into A8-35 was observed reproducibe and therefore appears to be specific to A8-35.

### Stability of purified ClC-rm1

**Fig 7:**
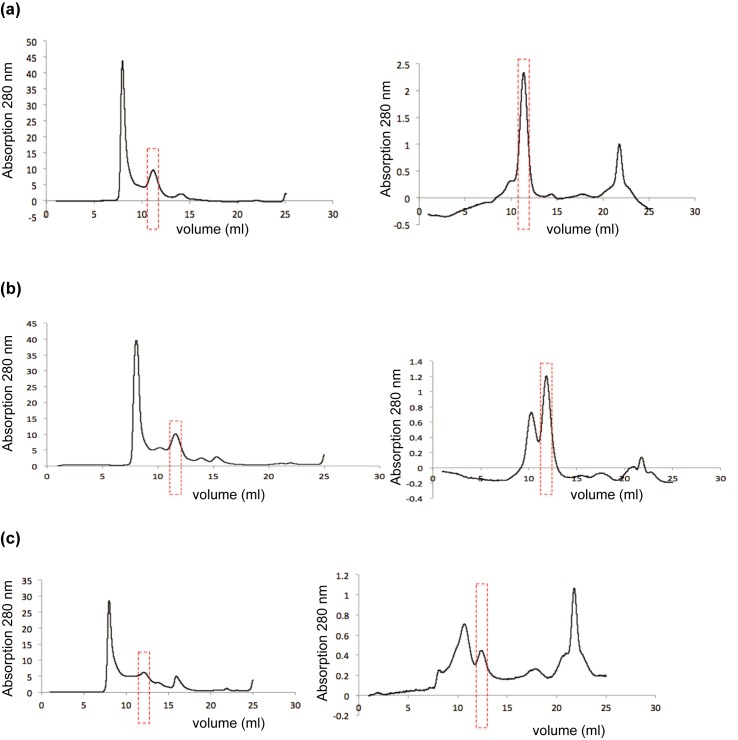
The stability of ClC-rm1 in different detergents was analyzed by SEC. (a) ClC-rm1 in DDM on day 0 (left panel) and after three days at 4°C (right panel). (b) ClC-rm1 on DM day 0 (left panel) and after three days at 4°C (right panel). (c) ClC-rm1 on OG day 0 (left panel) and after three days at 4°C (right panel). Dimeric ClC-rm1 fractions on SEC are highlighted by red dotted line rectangles.

High-resolution structural studies require that the protein sample remain stable throughout sample preparation. In general, membrane proteins are relatively unstable in solutions containing detergent, as they are significantly different from the native environment. We assessed the stability of ClC-rm1 samples in various detergent types over the course of three days. SEC fractions of dimeric ClC-rm1 in DDM, DM, and OG were kept at 4°C for three days and then re-assessed by SEC (Fig 7). ClC-rm1 in DDM was still largely monodisperse after the three-day, as judged by SEC (Fig 7A, right panel). SEC of ClC-rm1 in DM or OG after three days indicated significant accumulation of material in other fractions (Fig 7B and 7C, right), presumably representing degraded and aggregated materials.

### Contaminant elimination

We found that in each of the tested purification conditions, there was evidence of multiple contaminants (Figs 1B, 1D and 5B, labeled as “1”). Western blot analysis revealed that most of the bands were the monomeric and dimeric (oligomeric) ClC-rm1. However, further analysis by mass spectrometry revealed that both bands contained other proteins along with ClC-rm1. Table 1 summarizes the detected contaminants specifically in the DDM-purified ClC-rm1, ClC-m1 in A8-35, and DDM-purified ClC-rm1_eGFP.

**Table 1:** Mass spectrometry results of purified ClC-rm1 search against the *E*. *coli* protein data bank.

Sample name	# of Peptides	Sample name	# of Peptides
*Gel band ~58 kDa (ClC-rm1/DDM)*		*Gel band ~110 kDa (ClC-rm1/DDM)*	
ClC-rm1	116	ClC-rm1	85
atpD	11	acrB	54
arnA	10	aceE	11
hflB	9	arnA	11
kefC	5	atpD	8
trxA	3	hflB	8
atpA	3	tolC	8
lamB	3	yaeT	7
hflk	2	hflK	7
LF82-653	2	nuoG	7
YeaY	1	atpA	5
rho	1	ompF	5
pntA	1	glnD	4
		sucA	4
		Rne	4
		rplQ	3
		rplJ	3
		fadA	3
		dnaJ	3
		rplO	3
		trxA	2
		lldD	2
		gldA	2
		ftsZ	2
		tufB	2
		glpK	2
*Gel band ~58 kDa (ClC-rm1/A8-35)*		*Gel band ~110 kDa (ClC-rm1/A8-35)*	
ClC-rm1	48	ClC-rm1	7
atpD	22	acrB	111
tufB	16	aceE	16
hflB	11	CusA	12
acrB	12	arnA	9
cyoB	13	sucA	7
Rho	8	gyrA	6
Hflk	7	tolC	8
nuoF	5	ompF	5
cydA	5	secA	5
pntB	4	lamB	5
fruA	4	adhE	5
clpX	3	tufB	3
zltB	3	cyoB	2
alaS	3	alaS	2
gltA	3	Lpp	1
hemX	3	katE	1
glpK	2	glnE	1
arcA	2	dnaJ	1
gatZ	2	glmS	1
rmuC	2	carB	1
gldA	2	arnC	1
arnA	2	nmpC	1
msrA	2	rho	1
dnaK	1		
ftsZ	1		
cyoA	1		
Ogt	1		
aceF	1		
hslU	1		
yceG	1		
lpp	1		
Ndh	1		
Yqjl	1		
dkgA	1		
mukF	1		
rne	1		
hisB	1		
rtcR	1		
yoeB	1		
*Gel band ~80 kDa (ClC-rm1_eGFP/ DDM)*			
ClC-rm1_eGFP	415		
hflB	16		
GFP-Aequorea	291		
dnaK	10		
ompC	6		
ompF	5		
pplD	5		
yaeT	4		
dapB	3		
lamB	3		
tolC	3		
lepA	3		
sdhA	2		
dbpA	2		
Lmp	1		
lysC	1		
mopA	1		
mscS	1		
nuoC	1		
atpA	1		
aceF	1		
rpoC	1		
atpD	1		
rpsA	1		

One of the contaminants, the AcrB protein, is abundantly stable and has been reported as a contaminant in several structural studies of membrane proteins, including those purified using Ni^2+^ resins [4]. It has even been crystalized accidentally in some cases [33–36]. The co-purification is presumably due to the four intrinsic histidine residues at the AcrB C-terminus that can bind to Ni^2+^ resin similarly to engineered His tags. Previous studies suggest that AcrB contamination is particularly problematic when using maltose-based detergents such as DDM, DM, and LMNG [4]. As ClC-rm1 is a small membrane protein, it is important to remove small contaminants, such as AcrB, that have a similar appearance in electron microscope micrographs and can be mistaken for ClC-rm1. These contaminants will interfere with subsequent single-particle image processing.

**Fig 8:**
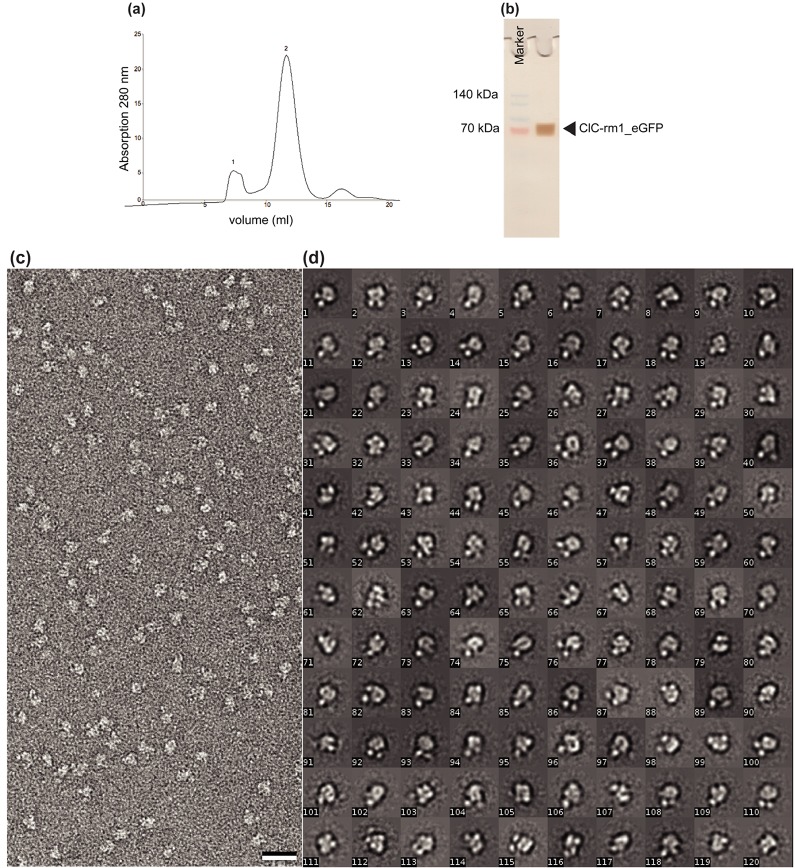
Elution profile of ClC-rm1_eGFP in DDM after superdex 200 SEC. Peak 1 corresponds to the void volume. Peak 2 corresponds to ClC-rm1_eGFP. (b) SDS-PAGE gel of ClC-rm1_eGFP after SEC. Lane 1: molecular mass marker. Lane 2 ClC-rm1_eGFP after SEC. ClC-rm1 monomer band is around ~80 kDa. (c) Micrograph of negatively stained ClC-rm1_eGFP particles. DDM-solubilized ClC-rm1_eGFP particles are mono-disperse. Scale bar 20 nm (d) Representative class averages of ClC-rm1_eGFP in DDM.

We implemented several strategies to eliminate the contaminants in purified ClC-rm1, including reducing the time spent in detergents for solubilization, optimizing the Ni^2+^ resin binding time, pre-washing the samples with a high concentration of imidazole, changing the expression medium (rich to minimal medium), and using alternative detergents. In our experiments, none of these strategies eliminated all contaminants. We then tried two strategies to minimize AcrB contamination while avoiding aggregation. The JW0451-2 *E*. *coli* strain was developed to prevent AcrB contamination [37] and others have shown that adding eGFP can promote proper folding abd increase the solubility of membrane protein during solubilization step [38–41]. Thus we express ClC-rm1 with eGFP fused to C-terminus in the AcrB knockout *E*. *coli* strain JW0451-2. After solubilization with DDM, ClC-rm1_eGFP protein was purified using either Ni^2+^ or Co^2+^ resins. Interestingly, ClC-rm1_eGFP bound to both types of resins. Though we found that ClC-rm1_eGFP bound with lower affinity to the Co^2+^ resins, we also found there to be fewer contaminant proteins after elution, making it preferable to Co^2+^ resin-based purification overall. Furthermore, SDS-PAGE analysis revealed extra bands in addition to ClC-rm1_eGFP following Co^2+^ resin purification. To further optimize the ClC-rm1_eGFP purification process, we reduced the solubilization and binding times. Following the optimized protocol (solubilization time; 30 min, and binding time; 30 min), purified ClC-rm1_eGFP showed a small aggregation peak (Superdex 200 SEC; Fig 8A) compared to the ClC-rm1 expressed in BL21(DE3) *E*. *coli* (Fig 2C, peak 1), confirming that eGFP-fused protein is less likely to aggregate.

**Fig 9:**
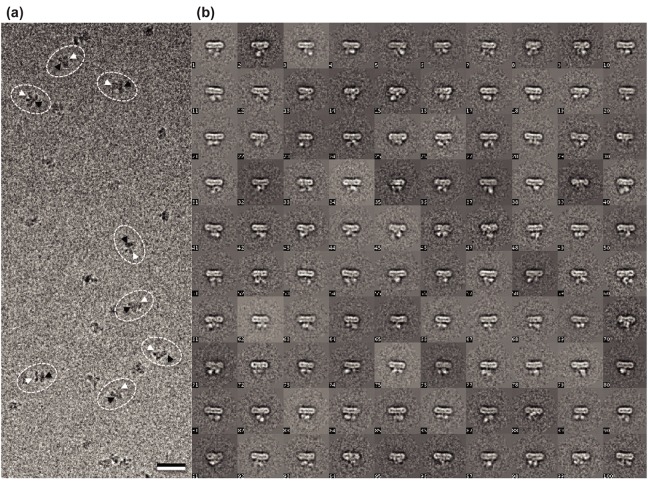
(a) Representative cryo-EM micrograph after motion correction. The black arrowhead indicates the transmembrane domain and white arrowhead indicates the cytoplasmic domain of ClC-rm1_eGFP particles. Scale bar 20 nm. (b) 2D class averages of cryo-EM ClC-rm1_eGFP particles.

We further analyzed the samples for purity and found that ClC-rm1_eGFP eluted from the superdex 200 column has a symmetric peak with molecular mass corresponding to the dimeric size of ClC-rm1_eGFP. SDS-PAGE analysis of ClC-rm1_eGFP after SEC showed a strong monomer band at ~80 kDa (Fig 8B). Mass spectrometry confirmed that the purified protein was indeed ClC-rm1. Electron micrographs of purified ClC-rm1_eGFP negatively stained with uranyl formate indicated monodisperse particles (Fig 8C). 29,300 particles were selected from 54 micrographs and classified into 300 classes. Representative class averages are shown in Fig 8D. Some classes showed two dot-like features that likely correspond to the cytoplasmic domains labelled with eGFP while the domain next to the dots must correspond to the transmembrane domain with a side length of ~115 Å, consistent with the side length of ClC-ec1 dimer [12]. Particles of ClC-rm1_eGFP on cryo micrographs (Fig 9A, highlighted in black and white arrow heads), as well as 2D class averages (Fig 9B) also showed clear densities corresponding to the transmembrane and cytoplasmic domains. Thus, our combined JW0451-2, *ΔacrB* ClC-rm1_eGFP expression approach effectively eliminated most contaminant proteins from the samples, including AcrB, while preserving structural integrity and minimizing aggregation. We believe this approach to be promising for preparing samples for high-resolution cryo-EM analysis.

## Conclusions

We conducted a systematic examination of several variables involved in the preparation of membrane protein samples for high-resolution structural studies including expression system, solubilization, purification and detergent exchange protocols, and assays for stability and impurity. We found that the detergent types was a key variable, as some detergents can contribute to contaminant co-purification [4]. Combining these considerations with techniques such as SEC and affinity resins can reduce the presence of contaminants. However, it often remains difficult to detect contaminant proteins before they are analyzed further for structural studies. When working with small membrane proteins, such as ClC-rm1, it is particularly important to remove all contaminants as even small molecules can interefere with cryo-EM structural studies. In these studies, we found that the small contaminant protein AcrB resisted purifictation by all standard methods. Thus, we turned instead to an expression system using the *E*. *coli* strain lacking AcrB (JW0451-2, *ΔacrB*). Additionally, we incorporated the eGFP to the C-terminus of ClC-rm1 as it promotes proper folding during expression of membrane proteins and increases the solubility during solubilization of membrane proteins. These strategies allowed us to express and purify properly folded, contaminant-free homogeneous ClC-rm1 protein suitable for high-resolution data collection. The presence of the eGFP tag will also help with the initial particle alignment as it adds clearly identifiable features to distinguish the cytoplasmic domian from the trnasmembrane domain of the protein. The tag may interfere with the alignment at high resolution due to its flexibility and density masking may have to be used to obtain accurate alignments. Our present study can serve as a guide when establishing new purification protocols, particularly of small membrane proteins, for cryo-EM and other quantitatiove structural studies.

## Supporting information

S1 Fig ClC-ec1 was over-expressed in BL21(DE3) *E*. *coli* cells and purified. The sample was 99% pure after eluting at pH 8 from the Co^2+^ resin. The purified sample was run on SEC and the protein was eluted as a single peak (left upper panel), then run as a single band on SDS-PAGE (right upper panel). Purified protein was confirmed by mass spectrometry. The corresponding molecular mass based on the SEC chromatogram, around 150 kDa, indicates the presence of ClC-ec1 dimers with associated detergent. An electron microscopy micrograph of purified ClC-ec1 negatively stained with uranyl acetate shows the presence of monodisperse and homogenous particles (lower panel). Scale bar 20 nm.
(EPS)

## References

[1] Drew D, Newstead S, Sonoda Y, Kim H, von Heijne G, Iwata S. GFP-based optimization scheme for the overexpression and purification of eukaryotic membrane proteins in Saccharomyces cerevisiae. Nat Protoc. 2008;3(5):784–98. doi: 10.1038/nprot.2008.44 18451787 10.1038/nprot.2008.44 PMC2744353

[2] Kawate T, Gouaux E. Fluorescence-detection size-exclusion chromatography for precrystallization screening of integral membrane proteins. Structure. 2006;14(4):673–81. doi: 10.1016/j.str.2006.01.013 16615909 10.1016/j.str.2006.01.013

[3] Parcej D, Guntrum R, Schmidt S, Hinz A, Tampe R. Multicolour fluorescence-detection size-exclusion chromatography for structural genomics of membrane multiprotein complexes. PLoS One. 2013;8(6):e67112 doi: 10.1371/journal.pone.0067112 23825631 10.1371/journal.pone.0067112 PMC3692423

[4] Wiseman B, Kilburg A, Chaptal V, Reyes-Mejia GC, Sarwan J, Falson P, et al Stubborn contaminants: influence of detergents on the purity of the multidrug ABC transporter BmrA. PLoS One. 2014;9(12):e114864 doi: 10.1371/journal.pone.0114864 25517996 10.1371/journal.pone.0114864 PMC4269414

[5] Maduke M, Pheasant DJ, Miller C. High-level expression, functional reconstitution, and quaternary structure of a prokaryotic ClC-type chloride channel. J Gen Physiol. 1999;114(5):713–22. 10539975 10.1085/jgp.114.5.713 PMC2230540

[6] Mindell JA, Maduke M, Miller C, Grigorieff N. Projection structure of a ClC-type chloride channel at 6.5 A resolution. Nature. 2001;409(6817):219–23. doi: 10.1038/35051631 11196649 10.1038/35051631

[7] van Heel M, Harauz G, Orlova EV, Schmidt R, Schatz M. A new generation of the IMAGIC image processing system. J Struct Biol. 1996;116(1):17–24. doi: 10.1006/jsbi.1996.0004 8742718 10.1006/jsbi.1996.0004

[8] Grant T, Grigorieff N. Measuring the optimal exposure for single particle cryo-EM using a 2.6 A reconstruction of rotavirus VP6. Elife. 2015;4:e06980 doi: 10.7554/eLife.06980 26023829 10.7554/eLife.06980 PMC4471936

[9] Rohou A, Grigorieff N. CTFFIND4: Fast and accurate defocus estimation from electron micrographs. J Struct Biol. 2015;192(2):216–21. doi: 10.1016/j.jsb.2015.08.008 26278980 10.1016/j.jsb.2015.08.008 PMC6760662

[10] Lucas M, Encinar JA, Arribas EA, Oyenarte I, Garcia IG, Kortazar D, et al Binding of S-methyl-5'-thioadenosine and S-adenosyl-L-methionine to protein MJ0100 triggers an open-to-closed conformational change in its CBS motif pair. J Mol Biol. 2010;396(3):800–20. doi: 10.1016/j.jmb.2009.12.012 20026078 10.1016/j.jmb.2009.12.012

[11] Abeyrathne PD, Chami M, Stahlberg H. Biochemical and biophysical approaches to study the structure and function of the chloride channel (ClC) family of proteins. Biochimie. 2016;128–129:154–62. 27554851 10.1016/j.biochi.2016.08.008

[12] Dutzler R, Campbell EB, Cadene M, Chait BT, MacKinnon R. X-ray structure of a ClC chloride channel at 3.0 A reveals the molecular basis of anion selectivity. Nature. 2002;415(6869):287–94. doi: 10.1038/415287a 11796999 10.1038/415287a

[13] Meyer S, Dutzler R. Crystal structure of the cytoplasmic domain of the chloride channel ClC-0. Structure. 2006;14(2):299–307. doi: 10.1016/j.str.2005.10.008 16472749 10.1016/j.str.2005.10.008

[14] Jentsch TJ, Stein V, Weinreich F, Zdebik AA. Molecular structure and physiological function of chloride channels. Physiol Rev. 2002;82(2):503–68. doi: 10.1152/physrev.00029.2001 11917096 10.1152/physrev.00029.2001

[15] Markovic S, Dutzler R. The structure of the cytoplasmic domain of the chloride channel ClC-Ka reveals a conserved interaction interface. Structure. 2007;15(6):715–25. doi: 10.1016/j.str.2007.04.013 17562318 10.1016/j.str.2007.04.013

[16] Meyer S, Savaresi S, Forster IC, Dutzler R. Nucleotide recognition by the cytoplasmic domain of the human chloride transporter ClC-5. Nat Struct Mol Biol. 2007;14(1):60–7. doi: 10.1038/nsmb1188 17195847 10.1038/nsmb1188

[17] Dutzler R. A structural perspective on ClC channel and transporter function. FEBS Lett. 2007;581(15):2839–44. doi: 10.1016/j.febslet.2007.04.016 17452037 10.1016/j.febslet.2007.04.016

[18] Seddon AM, Curnow P, Booth PJ. Membrane proteins, lipids and detergents: not just a soap opera. Biochim Biophys Acta. 2004;1666(1–2):105–17. doi: 10.1016/j.bbamem.2004.04.011 15519311 10.1016/j.bbamem.2004.04.011

[19] Kuhlbrandt W. Three-dimensional crystallization of membrane proteins. Q Rev Biophys. 1988;21(4):429–77. 3071823 10.1017/s0033583500004625

[20] Arnold T, Linke D. The use of detergents to purify membrane proteins. Curr Protoc Protein Sci. 2008;Chapter 4:Unit 4 8 1–4 8 30. 10.1002/0471140864.ps0408s53 18729050

[21] Prive GG. Detergents for the stabilization and crystallization of membrane proteins. Methods. 2007;41(4):388–97. doi: 10.1016/j.ymeth.2007.01.007 17367711 10.1016/j.ymeth.2007.01.007

[22] Rigaud J, Chami M, Lambert O, Levy D, Ranck J. Use of detergents in two-dimensional crystallization of membrane proteins. Biochim Biophys Acta. 2000;1508(1–2):112–28. 11090821 10.1016/s0005-2736(00)00307-2

[23] Abeyrathne PD, Arheit M, Kebbel F, castano-Diez D, Goldie KN, Chami M. Analysis of 2-D crystals of membrane proteins by electron microscopy.: Comprehensive Biophysics; 2012. 227–310 p.

[24] Yang Z, Wang C, Zhou Q, An J, Hildebrandt E, Aleksandrov LA, et al Membrane protein stability can be compromised by detergent interactions with the extramembranous soluble domains. Protein Sci. 2014;23(6):769–89. doi: 10.1002/pro.2460 24652590 10.1002/pro.2460 PMC4093953

[25] Bell SP, Curran PK, Choi S, Mindell JA. Site-directed fluorescence studies of a prokaryotic ClC antiporter. Biochemistry. 2006;45(22):6773–82. doi: 10.1021/bi0523815 16734414 10.1021/bi0523815

[26] Tribet C, Audebert R, Popot JL. Amphipols: polymers that keep membrane proteins soluble in aqueous solutions. Proc Natl Acad Sci U S A. 1996;93(26):15047–50. 8986761 10.1073/pnas.93.26.15047 PMC26353

[27] Liao M, Cao E, Julius D, Cheng Y. Structure of the TRPV1 ion channel determined by electron cryo-microscopy. Nature. 2013;504(7478):107–12. doi: 10.1038/nature12822 24305160 10.1038/nature12822 PMC4078027

[28] Chen Y, Clarke OB, Kim J, Stowe S, Kim YK, Assur Z, et al Structure of the STRA6 receptor for retinol uptake. Science. 2016;353(6302). 10.1126/science.aad8266 PMC5114850 27563101

[29] Zubcevic L, Herzik MA Jr., Chung BC, Liu Z, Lander GC, Lee SY. Cryo-electron microscopy structure of the TRPV2 ion channel. Nat Struct Mol Biol. 2016;23(2):180–6. doi: 10.1038/nsmb.3159 26779611 10.1038/nsmb.3159 PMC4876856

[30] Bai XC, Rajendra E, Yang G, Shi Y, Scheres SH. Sampling the conformational space of the catalytic subunit of human gamma-secretase. Elife. 2015;4. 10.7554/eLife.11182 PMC4718806 26623517

[31] Popot JL, Althoff T, Bagnard D, Baneres JL, Bazzacco P, Billon-Denis E, et al Amphipols from A to Z. Annu Rev Biophys. 2011;40:379–408. doi: 10.1146/annurev-biophys-042910-155219 21545287 10.1146/annurev-biophys-042910-155219

[32] Postis V, Rawson S, Mitchell JK, Lee SC, Parslow RA, Dafforn TR, et al The use of SMALPs as a novel membrane protein scaffold for structure study by negative stain electron microscopy. Biochim Biophys Acta. 2015;1848(2):496–501. doi: 10.1016/j.bbamem.2014.10.018 25450810 10.1016/j.bbamem.2014.10.018 PMC4331651

[33] Glover CA, Postis VL, Charalambous K, Tzokov SB, Booth WI, Deacon SE, et al AcrB contamination in 2-D crystallization of membrane proteins: lessons from a sodium channel and a putative monovalent cation/proton antiporter. J Struct Biol. 2011;176(3):419–24. doi: 10.1016/j.jsb.2011.09.005 21964467 10.1016/j.jsb.2011.09.005

[34] Psakis G, Polaczek J, Essen LO. Acr B et al: Obstinate contaminants in a picogram scale. One more bottleneck in the membrane protein structure pipeline. J Struct Biol. 2009;166(1):107–11. doi: 10.1016/j.jsb.2008.12.007 19162196 10.1016/j.jsb.2008.12.007

[35] Veesler D, Blangy S, Cambillau C, Sciara G. There is a baby in the bath water: AcrB contamination is a major problem in membrane-protein crystallization. Acta Crystallogr Sect F Struct Biol Cryst Commun. 2008;64(Pt 10):880–5. doi: 10.1107/S1744309108028248 18931428 10.1107/S1744309108028248 PMC2564894

[36] Kalbermatter D, Jeckelmann JM, Chiu PL, Ucurum Z, Walz T, Fotiadis D. 2D and 3D crystallization of the wild-type IIC domain of the glucose PTS transporter from Escherichia coli. J Struct Biol. 2015;191(3):376–80. doi: 10.1016/j.jsb.2015.08.003 26260226 10.1016/j.jsb.2015.08.003

[37] Baba T, Ara T, Hasegawa M, Takai Y, Okumura Y, Baba M, et al Construction of Escherichia coli K-12 in-frame, single-gene knockout mutants: the Keio collection. Mol Syst Biol. 2006;2:2006 0008. 10.1038/msb4100050 PMC1681482 16738554

[38] Geertsma ER, Groeneveld M, Slotboom DJ, Poolman B. Quality control of overexpressed membrane proteins. Proc Natl Acad Sci U S A. 2008;105(15):5722–7. doi: 10.1073/pnas.0802190105 18391190 10.1073/pnas.0802190105 PMC2311375

[39] Schlegel S, Hjelm A, Baumgarten T, Vikstrom D, de Gier JW. Bacterial-based membrane protein production. Biochim Biophys Acta. 2014;1843(8):1739–49. doi: 10.1016/j.bbamcr.2013.10.023 24200679 10.1016/j.bbamcr.2013.10.023

[40] Drew D, Lerch M, Kunji E, Slotboom DJ, de Gier JW. Optimization of membrane protein overexpression and purification using GFP fusions. Nat Methods. 2006;3(4):303–13. doi: 10.1038/nmeth0406-303 16554836 10.1038/nmeth0406-303

[41] Drew D, Slotboom DJ, Friso G, Reda T, Genevaux P, Rapp M, et al A scalable, GFP-based pipeline for membrane protein overexpression screening and purification. Protein Sci. 2005;14(8):2011–7. doi: 10.1110/ps.051466205 15987891 10.1110/ps.051466205 PMC2279312

